# *In vivo* Multiphoton Microscopy Technique to Reveal the Physiology of the Mouse Placenta

**DOI:** 10.1111/j.1600-0897.2012.01161.x

**Published:** 2012-05-24

**Authors:** Ana C Zenclussen, David N Olivieri, Michael L Dustin, Carlos E Tadokoro

**Affiliations:** 1 Department of Experimental Obstetrics and Gynecology, Medical Faculty, Otto-von-Guericke UniversityMagdeburg, Germany; 2 Escuela Superior de Ingeniería Informatica, University of VigoVigo, Spain; 3 Skirball Institute of Biomolecular Medicine, New York University School of MedicineNew York, NY, USA; 4 Laboratory of Immune Regulation, Instituto Gulbenkian de CiênciaOeiras, Portugal

**Keywords:** Intravital imaging, malaria, placenta, *Plasmodium chabaudi*, two-photon microscopy

## Abstract

**Problem:**

Pregnancy is a challenge to the maternal immune system as it must defend the body against pathogens while at the same time develop immune tolerance against the fetus growing inside the uterus. Despite *ex vivo* techniques being used to understand these processes, *in vivo* techniques are missing.

**Method of Study:**

To directly study these phenomena, we have developed a new microscope stage and surgical procedures for use in two-photon microscopy, for *in vivo* observation of the mouse placenta.

**Results:**

These tools and surgical procedures demonstrate fetal and maternal blood flow inside the labyrinth zone of the placenta, as well as its three dimensional structure. It was also useful to identify *Plasmodium chabaudi*-infected red blood cells inside this labyrinth zone.

**Conclusion:**

We believe this technique will represent an important contribution for expanding the available knowledge concerning cell dynamics and interactions at the fetal-maternal interface.

## Introduction

The immune system evolved to maintain the integrity and function of host tissues. It is composed of different types of cells, each exerting specialized functions at different levels and operating under tight regulation.^1^ When this regulation breaks down, the function of targeted organs can fail leading to system-wide collapse.^2^ As an example, an imbalanced immune response that targets the reproductive system could lead to a miscarriage or impaired fetal development.^3^ Such events, if widespread, could obviously compromise the future of the entire species.

Pregnancy may be considered a challenge to the maternal immune system, which must maintain an effective defense against pathogens, while at the same time, develop immune tolerance against the semi-allogeneic fetus growing within the uterus.^4^ Indeed, the fetal tissue is in direct contact with maternal blood containing immune cells having the potential to destroy tissues bearing foreign antigens.^5^ Contrary to the initial theories positing immune ignorance against paternal antigens, it is now widely known that fetal structures are actively tolerated rather than ignored.^4^

During pregnancy, the potential for maternal infection adds further complexity to the functioning of the immune system. Depending upon the nature and severity of the infection, pregnancy can abruptly end with abortion or pre-term birth; the latter leading to serious consequences for both mother and baby and high healthcare costs for the premature neonates.^6^ In this context, an acute example is the maternal infection with *Plasmodium* sp. parasites, which can cause a pathology called ‘placental malaria’ (PM),^7^ which can be recreated and more intensely studied in a mouse model. According to recent studies of PM in mice, it has been hypothesized that *Plasmodium berghei*-infected red blood cells (Pb-iRBCs) could stop flowing within the placenta, thereby compromising oxygen and nutrient flow from mother to fetus.#b^8^b^9^ Nonetheless, the effect of parasite presence before pregnancy or in the early stages of embryo development remains to be clarified.

Thus, the development of new intravital imaging techniques that can provide more accurate and detailed *in situ* cell and parasite identification is necessary to reveal the behavior of such cells within different physiological and pathological situations. Also, these techniques lead to the development of future diagnostic and therapeutic strategies for the treatment of reproductive pathologies. Consistent with this goal, we describe here a novel technique that provides microscopic observations within the placenta, suggesting the utility of this new method.

## Materials and methods

### Animals and Parasites

In this study, we used C57Bl/10.PL (B10.PL) and B10.PL.Cyan animals (animals expressing Cyan protein under the ubiquitin promoter). All animals were produced in IGC animal facility and used after 6 weeks old. Infected red blood cells (iRBCs) were harvested from blood of *Plasmodium chabaudi*-GFP-infected animals between days 7 and 10 of infection. The percentage of iRBCs was approximately 40–50%. All procedures were approved by the Institutional Animal Care and Use Committee (IACUC), and they were in agreement with the Federation of European Laboratory Animal Science Associations (FELASA) directives, approval ID number AO10/2010.

### *P. chabaudi-*iRBC Injection

Female B10.PL mice were injected intravenously with blood containing red blood cells infected with *P. chabaudi*-GFP^10^ with the percentage described above. To avoid the coagulation of transferred blood into the recipient, 200 μL of infected blood was mixed with 700 μL of PBS and 100 μL of rhodamine B isothiocyanate-dextran 70,000 KDa (stock at 20 mg/mL; Sigma-Aldrich, Inc., St. Louis, MO, USA). After removal of coagulum, the remaining suspension (400–500 μL) was slowly injected into the recipients. The placental images were acquired 20–30 min after this injection using a two-photon microscope, as described in more detail later in this section.

### Determination of Estrus Cycle Phases

We performed a histological examination of vaginal smears from cells harvested after a vaginal lavage to check the exact phase of the estrus cycle. Briefly, each female vagina was washed with 50 μL of PBS by gentle pipetting. These cells were centrifuged at 400 ***g*** for 5 min, and the pellet resuspended in 10 μL of PBS. This volume was then spread over a glass slide and, after drying, each smear was fixed in 100% methanol. When the alcohol evaporated, each slide was stained with Giemsa for 1 hr at room temperature. After this dyeing period, each slide was washed in tap water and reserved for future microscopic examination.

### Generation of Pregnant Females for Imaging of *P. chabaudi*-iRBCs

Pregnant B10.PL females (crossed with B10.PL males) were prepared for image acquisition between gestational days 15 and 17 of pregnancy. For maternal blood vessels and iRBC observation, each animal was injected with rhodamine B isothiocyanate-dextran and GFP^+^ iRBCs as described previously. To be able to simultaneously observe the maternal and fetal blood flow, different types of fluorescent-labeled Dextrans were injected in maternal or fetal circulation.

### Immunofluorescence of *P. chabaudi*-iRBCs Inside Mouse Placentas

Pregnant B10.PL females (crossed with B10.PL. Cyan males) between gestational days 15 and 17 of pregnancy were injected with GFP^+^ iRBCs as described previously. Thirty to Sixty minutes after injection, the animals were sacrificed, and the placentas were removed, transversally cut, and frozen in O.C.T. freezing media. Each frozen block was cut in a cryostat machine (Leica CM3050) at 8-μm thickness. Each slide was fixed in 1% PFA, for 30 min at R.T., and labeled with DAPI (5 ng/mL), 5 min at R.T., for nuclear staining. After 3 × 5 min washes in PBS, these slides were mounted using Fluoromount G (Southern Biotech, Birmingham, AL, USA). Since these females were crossed with Cyan males, fetal-origin tissues were also Cyan^+^. The images were acquired in fluorescence microscope (Leica DMRA2) using MetaMorph software (Molecular Devices, Woburn, MA, USA).

### Preparation of Animals for Placenta Imaging Acquisition

Animals were anesthetized by i.p. injection of ketamine (120 μg/g of mouse weight)/xylazine (16 μg/g of mouse weight) and kept on top of a heating pad at 37°C. After the animals were unconscious, a 3-cm skin incision was made on the lateral side of the abdomen, and subsequently, the underside portion of the peritoneal membrane was cut. One of the uterine horns was carefully exposed, avoiding any blood vessel disruption. The uterine wall was cut, revealing the individual amniotic sacs, each containing one fetus and the respective placenta. Each amniotic sac was cut and the placentas exposed, keeping the fetal umbilical cords intact. PBS-soaked cotton covered the fetuses to avoid dehydration. A small aluminum foil clip was used to hold one of the placentas. This step is crucial and has to be handled with extreme care to avoid placental detachment. Low-melting agarose of 2% (NuSieve GTG agarose; Lonza Inc., Allendale, NJ, USA) was placed on top of the preparation, and the whole system was closed by a top heater with a hole in the middle. At this point, the animal received (or not) 100 μL of rhodamine B isothiocyanate-dextran to allow blood flow observation. Next, we transferred the whole animal to the animal stand, where the microscope stage was positioned to stabilize the aluminum foil clip and to support the top tissue heater. After this final assembly, the whole preparation was moved to the microscope.

### Stereoscope Image Acquisition of the Placenta Blood Vessels

Animals, prepared as described above, were used to image the entire placenta vasculature structure using a Zeiss Stereo Lumar stereoscope (Zeiss, Inc., Chester, VA, USA), in an Apo Lumar S1.2× FWD 47-mm objective. Images were acquired using micro-manager v.1.3 software, and the magnifications are described elsewhere. To allow fetal blood observation, the placenta-corresponding embryo was intraperitoneally injected with 40 μL of FITC-Dextran 2,000,000 Da (Sigma-Aldrich Inc.) just before transferring the whole preparation to a stereoscope or microscope. Sequential images were acquired in the stereoscope to allow observation of fetal blood flow.

### Two-Photon Microscope Image Acquisition of the Placentas

Our two-photon microscope is equipped with a Chameleon Ti:Sapphire laser (Coherent, Inc., Santa Clara, CA, USA), four top PMTs for simultaneous up to four channel acquisitions, and a 20× water immersion objective (Olympus Inc., Center Valley, PA, USA). We first used a 565-nm dichroic mirror to diverge the incoming fluorescence into two pairs of PMTs. Also, we used two other Olympus-BX2 Chroma holders to diverge different wavelengths. One holder contained a 495-nm dichroic mirror, a 525/50-nm filter (for iRBC-GFP or FITC-Dextran signals), and a 460/50-nm filter (for Cyan signal). The second holder contained a 640-nm dichroic mirror, a 595/50-nm filter (for dextran-rhodamine B signal), and a 660/40-nm filter (not used). The combination of the detected wavelengths depends upon the different experimental setups and is specified in the figure legends. Sequential images of a 50-μm-depth tissue volume, divided into 4.0-μm z-steps constituting the 5D (x, y, z, t, and color), were acquired to allow observation of iRBC-GFP inside placental blood vessels. Each acquisition volume takes approximately 30 s to be scanned by the laser microscope. Some females were crossed with B10.PL. Cyan males to allow fetal tissue observation. z-stack images of the placentas were acquired to allow 3D reconstruction. We used imaris software (Bitplane AG, Inc., South Windsor, CT, USA) for constructing three-dimensional rendered models.

## Results

### Intravital Imaging of the Placenta

After successful conception, the blastocyst attaches into the uterine wall, giving rise to decidualization and, subsequently, to placentation. The development of a functional placenta is required for the maintenance of the pregnancy and fetal development, as the fetus depends entirely upon the placenta for nutrition and waste elimination. Indeed, the placenta is a unique and highly complex transient organ with specialized areas that allows the maximal interchange of substances between mother and fetus. The murine (and human) placenta is in direct contact with fetal trophoblasts, or hemochorionic,^11^ and is divided between fetus and mother with several anatomical structures: the chorionic plate, the labyrinth zone (where the labyrinth trophoblast cells make the walls of this maze), the junctional zone (containing spongiotrophoblasts, glycogen cells, and a layer of trophoblast giant cells), and a layer of maternal decidual cells.#b^12^b^13^b^14^ The labyrinth zone is responsible for the exchange of nutrients and gases between mother and fetus (Fig. S1a,b), reaching a maximum interchange on day 16.5 of gestation,^14^ while the junctional zone is in charge of producing important metabolites such as decidual prolactin-related proteins. Giant cells are thought to be important for implantation; however, strong evidence for this hypothesis is lacking.^15^ For this reason, the *in vivo* observation of both the placenta structures and the immune cell dynamics is essential for a better understanding of the fetal-maternal interface.

**Fig. 1:**
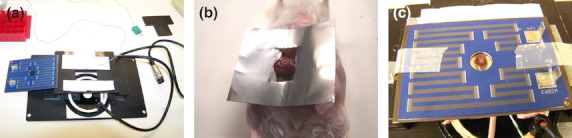
Pictures of placenta preparations. (a) bottom and top heaters (blue rectangle and black round pieces), organ stage holder (black metal frame), and heating probe (small green-white wire); (b) placenta exposed showing the aluminum foil clip; (c) final assembly of placenta preparation.

**Fig. 2:**
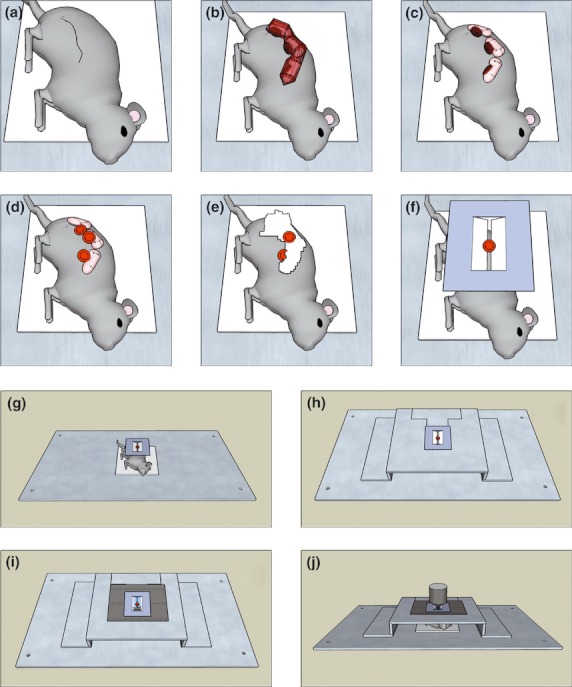
Representation of surgical procedure and stage for intravital imaging acquisition of placentas. (a) The pregnant mouse was deeply anesthetized, put on top of a heating pad, and a 3-cm skin incision was made on the lateral side of the abdomen. (b) The underneath peritoneal membrane was also cut after this. One of the uterus branches was carefully exposed, avoiding any blood vessel disruption. (c) The uterine wall was cut, revealing the individual amniotic sacs, each containing one fetus and the respective placenta. (d) Each amniotic sac was cut and the placentas exposed, keeping the fetal umbilical cords intact. (e) PBS-soaked cotton covered the fetuses to avoid dehydration. (f) A small aluminum foil clip was used to hold one of the placentas. This step is crucial and has to be handled with extreme care to avoid placental detachment. (g) Transfer of the whole animal to the animal stand. (h) Where the ‘stereotactic’ holder was positioned to stabilize the aluminum foil clip. (i) To support the top tissue heater. (j) After this final assembly, the whole preparation was moved to the microscope, here represented by the objective only.

**Fig. 3:**
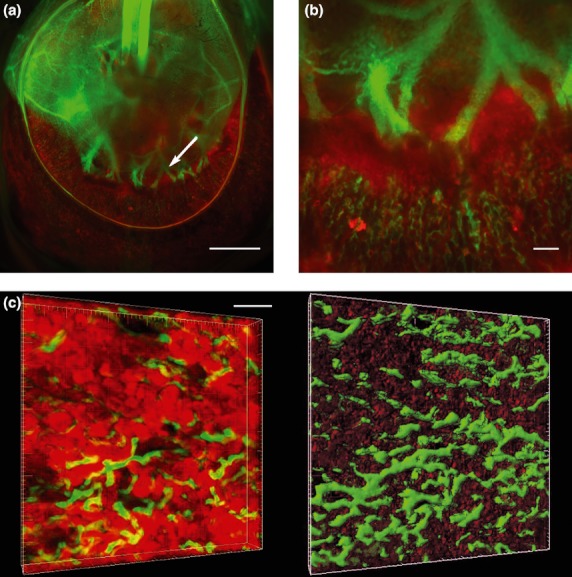
Intravital images of different placenta areas. The pregnant mouse was surgically prepared as described in Figs 1 and 2. (a) The chorionic plate can be easily identified in the fetal face of the placenta (white scale bar = 1 mm). The arrow indicates the region where the chorionic plate border is thinner. The umbilical cord is located in the top-middle of the field. (b) The labyrinth zone can be imaged at the border of the chorionic plate or underneath the chorionic plate after its removal (white scale bar = 100 μm). Note the mother blood spread inside this labyrinth. (c) Two-photon microscopy of this preparation allows the identification of maternal (injected with rhodamine B isothiocyanate-dextran) and fetal (injected with FITC-Dextran) blood vessels (left side, white scale bar = 100 μm), and their future 3D reconstruction (right side). Excitation laser wavelength: 820 nm; FITC-Dextran emission: 519 nm; rhodamine B isothiocyanate-dextran: 600 nm. These results are representative of a total of three distinct experiments.

**Fig. 4:**
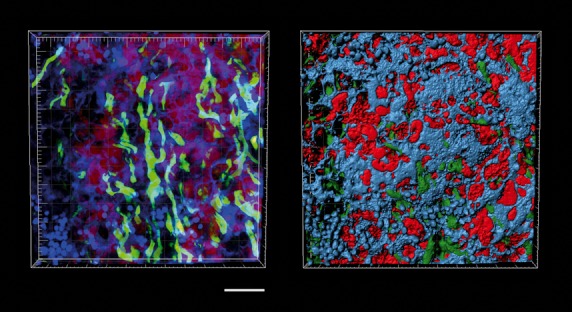
Tri-dimensional placenta reconstruction. The pregnant mouse was surgically prepared as described in Figs 1 and 2. On the left side, two-photon microscopy showing maternal (injected with rhodamine B isothiocyanate-dextran) and fetal (injected with FITC-Dextran) blood vessels. Since this female was crossed with a B10.PL. Cyan male, fetal tissue (blue) can also be observed; on the right side, a 3D reconstruction of this region. White scale bar = 100 μm. Excitation laser wavelength: 820 nm; FITC-Dextran emission: 519 nm; rhodamine B isothiocyanate-dextran: 600 nm; Cyan+ cells emission: nm. These results are representative of a total of three distinct experiments.

Imaging of the placenta is depicted in Fig. 1 and Fig. 2. One key manipulation in the preparation is with regard to the placenta handling during clipping, which requires great care. The entire structure is connected only through the *decidua basalis* region, where the larger maternal blood vessels reach the tissue. Any abrupt movement of this area may cause a placental detachment and hemorrhage, leading to a sudden cessation in blood flow to the fetus and death to the mother by exsanguination. It is also important to note here that compression of placenta against the glass slide on top of preparation can disturb the normal placental blood flow, which could lead to artifacts in terms of cellular adhesion and interactions between blood and trophoblast cells. Once the entire placenta is exposed, with fetal side-up, the chorionic plate can easily be imaged, because it is completely exposed in this orientation (Fig. 3a). The labyrinth zone is located in the marginal areas around the chorionic plate. The surrounding chorionic sac can be removed from the area by gently moving the sac to one side using fine tweezers, as we did in our preparations (Fig. 3b). The fetal circulation was intact for at least 1 hr based upon imaging of blood flow after intra-embryonic FITC-Dextran injection (Video S1). Two-photon microscopy was used for 3D reconstruction of all the placental layers (Fig. 3c, Fig. 4, and Videos S2 and S3) and can help to decipher immune cell dynamics in the placenta under normal and pathological conditions.

### *Plasmodium chabaudi*-iRBC Inside Placentas

**Fig. 5:**
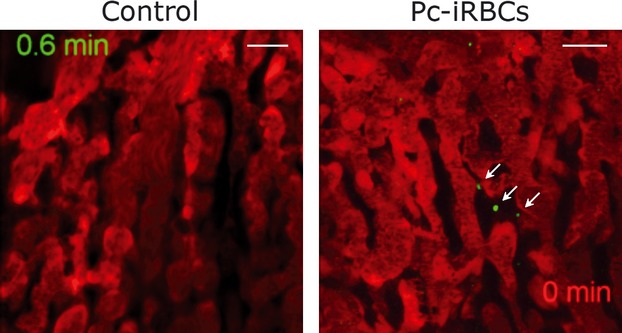
*Plasmodium chabaudi*-iRBC accumulation inside placental labyrinth. Placentas were prepared as described and images acquired in a two-photon microscope, before (Control) and after the i.v. injection of red blood cells infected with *P. chabaudi*-GFP (Pc-iRBCs). White scale bar = 100 μm. Excitation laser wavelength: 900 nm; GFP wavelength emission: 509 nm; rhodamine B isothiocyanate-dextran: 600 nm. These results are representative of a total of three distinct experiments.

Placental malaria is a model for compromised placental function during infection. It has been suggested that Pb-iRBCs are sequestered within the placental labyrinth.^9^ Here, we used *Plasmodium chabaudi*-iRBCs (Pc-iRBCs) for *in vivo* observation of Pc-iRBCs inside the mouse placenta. To identify these Pc-iRBCs inside the placenta, we used *P. chabaudi* expressing GFP; these GFP^+^ iRBCs can easily be found in placenta slices (Fig. S1c). Then, we used two-photon microscopy to acquire images of blood flow inside the placenta before (Video S4) and after intravenous injection of Pc-iRBCs (Video S5). From a comparison with the established baseline for normal maternal blood flow, we could observe a small accumulation of Pc-iRBCs inside the placenta labyrinth during infection (Fig. 5). Thus, further application of this technology should provide a deeper understanding of placental changes during infection that could have profound influence upon future embryo development.

## Discussion

Mammalian evolution may have been greatly influenced by the uterus' viviparous capacity and the existence of an intrauterine structure for embryo nurturing during development. This may be particularly true as both uterine and placental Metatheria (marsupials) and Eutheria (other mammals) animals outnumber the species of Prototheria animals, represented today only by platypus and echidnas. Moreover, many maternal physiologic systems and structures, as well as the immune system, have adapted to pregnancy. The immune system evolved to allow the growth of a semi-allogeneic tissue represented by the embryo through the active generation of tolerogenic processes; these mechanisms include the development of cells with the specific capacity to suppress the immune system such as regulatory T cells (Tregs). This is particularly fascinating, considering that the maternal immune system tolerates and fosters the growth and well-being of the growing fetus, while at the same time protecting the mother from infections. Nonetheless, this finely regulated balance can be compromised in the case of acute infections, leading possibly to premature pregnancy termination or fetal loss.

In this work, we establish a method for the application of two-photon microscopy to the placenta. For this, it is important to first study the placenta under non-infected conditions. Thus, our intravital microscopy preparation was used to observe the fetal blood flow by injecting different fluorescent dyes in pregnant mice and their fetuses (Video S1) and reconstructing the ‘spongy’ placental labyrinth layer (Fig 3c, Fig. 4, and Videos S2 and S3). This technique confirms the vast knowledge we have concerning mouse placenta histology and is therefore an excellent tool for studying blood flow within the placenta during infectious pathologies. An important placental pathology is placental malaria,^8^ where the existence of direct sequestration of iRBCs inside the living placenta is a central question. By using our intravital image acquisition technique of placentas from mice injected intravenously with Pc-iRBCs, it has confirmed that iRBCs could easily be detected (Video S5). Indeed, our data show that these iRBCs accumulate within certain regions of the tissue (Fig. 5) and could provide insight into designing novel approaches to clear the iRBC from the placenta. In this case, our intravital technique could be used to further elucidate the behavior of iRBCs in PM models.

In conclusion, we have developed a novel microscope stage and surgical procedure that allow *in vivo* observation of the fetal-maternal interface by the use of a two-photon microscope. This method should be useful for researchers in the field of reproductive biology, immunology, and parasitology. We are confident that this protocol will greatly improve our understanding of *in vivo* immunological events that occur during placentation and the effect of concomitant infections to the success of pregnancies.

## Supporting Information

**Video S1.** Fetal blood flow inside the placenta.

**Video S2.** 3D reconstruction of maternal-fetal blood vessels inside the placenta.

**Video S3.** 3D reconstruction of placenta.

**Video S4.** Maternal blood flow inside the placenta.

**Video S5.** *Plasmodium chabaudi*-infected red blood cells (Pc-iRBCs) stop flowing inside the placenta.

**Figure S1.** Representation of mouse placenta and *Plasmodium chabaudi*-GFP. This diagram provides a clear understanding of the entire structure of the mouse placenta labyrinth area. (a) A representation of the placenta in a lateral/cross section view; the fetus, umbilical cord, labyrinth zone, and deciduas area, where the maternal blood vessels (red) in this cross section view of the labyrinth area are cut transversally. (b) A top view of the same labyrinth area where the maternal (red) and fetal (green) blood vessels can be seen in a longitudinal section; which is the view provided in our placenta movies. (c) P. chabaudi expressing GFP infects red blood cells (RBCs) and these infected RBCs can be identified within the placenta by GFP expression (green); red: DAPI staining; blue: CFP expression by fetal tissues; white bar = 10μm; the arrow shows one GFP-iRBC.
