# Supplementary material for: In vivo Multiphoton Microscopy Technique to Reveal the Physiology of the Mouse Placenta
Source: Am J Reprod Immunol. 2012 May 24;68(3):271–8. doi: 10.1111/j.1600-0897.2012.01161.x (PMC3465783; doi:10.1111/j.1600-0897.2012.01161.x)
Supplement: Supplementary file 6 [file aji0068-0271-SD7.pdf]

Supplemental videos:

Supplemental video 1: **Fetal blood flow inside the placenta**. Day 17 pregnant female B10.PL mice were deeply anesthetized and one of the placentas exposed, according to the protocol summarized in figure 2. Fetuses were i.p. injected, underneath the umbilical cord, with FITC-Dextran just before the image acquisition. The image was acquired in a Zeiss Stereo Lumar stereoscope. A total of 150 consecutive tiff images were acquired during 5 min. The fluorescent lamp was kept on during the whole period of observation and the filters used were: excitation: BP 470/40 nm; emission: BP525/50 nm. This movie is representative of 3 independent observations in total. Note the fetal blood flow throughout the placental labyrinth zone and umbilical cord.

Supplemental video 2: **3D reconstruction of maternal-fetal blood vessels inside the placenta**. Day 17 pregnant female B10.PL mice were deeply anesthetized and one of the placentas exposed, according to the protocol summarized in figure 2. Fetuses were i.v. injected (underneath the umbilical cord) with FITC-Dextran and mothers were i.v. injected with Rhodamine B-Dextran. Both injections were done just before the image acquisition. Z-stack images were acquired in a two-photon microscope. The final 3D reconstruction was done in Imaris software. These images were acquired using the following configuration: excitation laser wavelength: 840 nm; FITC-Dextran emission: 519 nm; Rhodamine B isothiocyanate-Dextran: 600 nm. This movie is representative of 3 observations in total. This 3D reconstruction shows the distribution of fetal (green) and maternal (red) blood, first as observed and after as a 3D model.

Supplemental video 3: **3D reconstruction of placenta**. Day 17 pregnant female B10.PL mice were deeply anesthetized and one of the placentas exposed, according to the protocol summarized in figure 2. Fetuses were i.v. injected (underneath the umbilical cord) with FITC-Dextran and mothers were i.v. injected with Rhodamine B-Dextran. Both injections were done just before the image acquisition. To allow fetal trophoblasts observation, these females were crossed with B10.PL.Cyan males. Z-stack images were acquired in a two-photon microscope. The final 3D reconstruction was done in Imaris software. These images were acquired using the following configuration: excitation laser wavelength: 840 nm; FITC-Dextran emission: 519 nm; Rhodamine B isothiocyanate-Dextran: 600 nm; Cyan emission: 480nm. This movie is representative of 3 observations in total. This 3D reconstruction shows the distribution of fetal tissues (blue), fetal (green) and maternal (red) blood; it shows first as observed and after as a 3D model.

Supplemental video 4: **Maternal blood flow inside the placenta**. Day 17 pregnant female B10.PL mice were deeply anesthetized and one of the placentas exposed, according to the protocol summarized in figure 2. Each animal were i.v. injected with

Rhodamine B-Dextran just before the image acquisition. Sequential z-stack images were acquired in a two-photon microscope by a period of 15 min. These images were acquired using the following configuration: excitation laser wavelength: 900 nm; Rhodamine B isothiocyanate-Dextran: 600 nm. This movie is representative of 3 observations in total. Note the maternal blood flowing inside the placental labyrinth zone.

Supplemental video 5: ***P. chabaudi*-infected red blood cells (Pc-iRBCs) stop flowing inside the placenta.** Day 17 pregnant female B10.PL mice were deeply anesthetized and one of the placentas exposed, according to the protocol summarized in figure 2. Each animal were i.v. injected with Rhodamine B-Dextran containing Pc-iRBCs just before the image acquisition. Sequential z-stack images were acquired in a two-photon microscope by a period of 25 min. The final movie was done in ImageJ software. These images were acquired using the following configuration: excitation laser wavelength: 900 nm; GFP wavelength emission: 509 nm; Rhodamine B isothiocyanate-Dextran: 600 nm. This movie is representative of 3 observations in total. Note the GFP+ Pc-iRBCs stopped at the central region of the observed field.
