# Supplementary figures and images for: In vivo Multiphoton Microscopy Technique to Reveal the Physiology of the Mouse Placenta
Source: Am J Reprod Immunol. 2012 May 24;68(3):271–8. doi: 10.1111/j.1600-0897.2012.01161.x (PMC3465783; doi:10.1111/j.1600-0897.2012.01161.x)

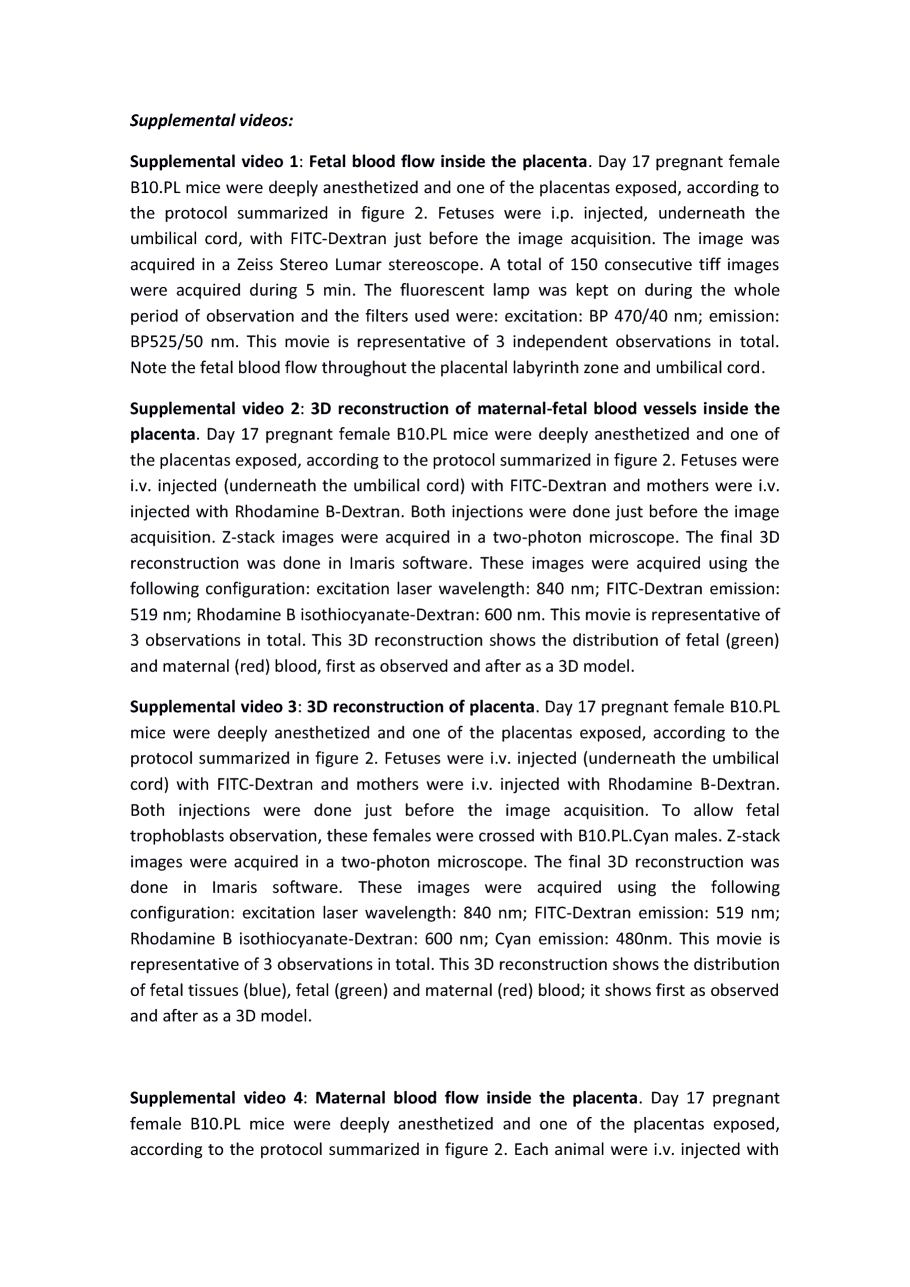

Supplement: Supplementary file 7 [file aji0068-0271-SD8.png]

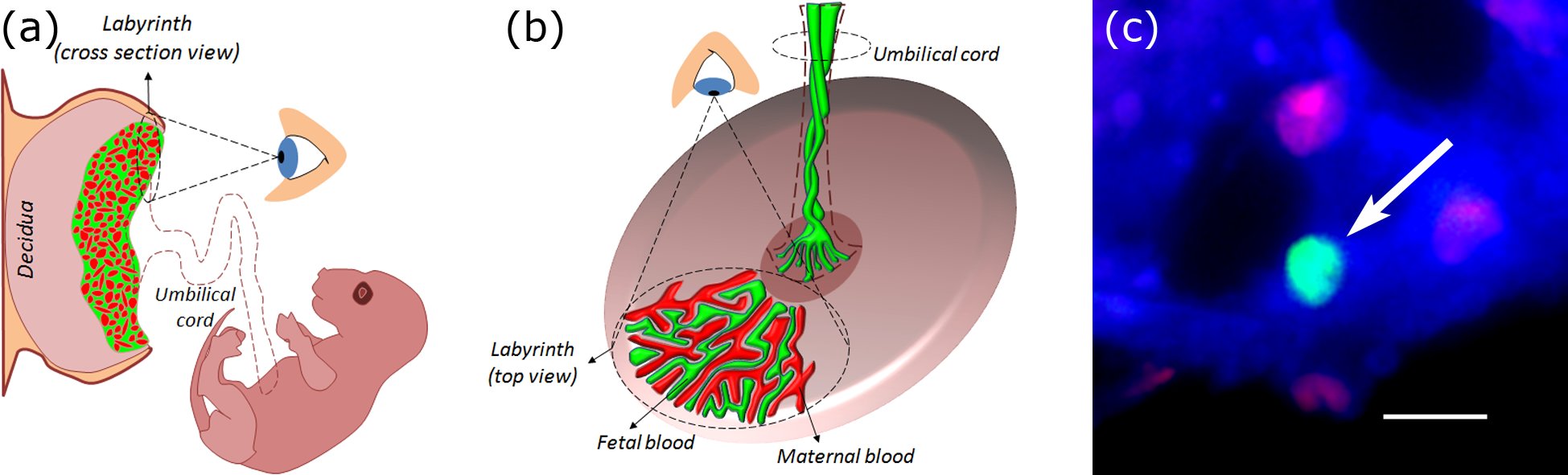

Supplement: Supplementary file 9 [file aji0068-0271-SD6.jpg]
